# Primate-specific evolution of an *LDLR* enhancer

**DOI:** 10.1186/gb-2006-7-8-r68

**Published:** 2006-08-02

**Authors:** Qian-fei Wang, Shyam Prabhakar, Qianben Wang, Alan M Moses, Sumita Chanan, Myles Brown, Michael B Eisen, Jan-Fang Cheng, Edward M Rubin, Dario Boffelli

**Affiliations:** 1 Genomics Division, Lawrence Berkeley National Laboratory, Berkeley, California 94720, USA; 2 US Department of Energy Joint Genome Institute, Walnut Creek, California 94598, USA; 3 Dana-Farber Cancer Institute, Harvard Medical School, Boston, Massachusetts 02115, USA

## Abstract

**Background:**

Sequence changes in regulatory regions have often been invoked to explain phenotypic divergence among species, but molecular examples of this have been difficult to obtain.

**Results:**

In this study we identified an anthropoid primate-specific sequence element that contributed to the regulatory evolution of the low-density lipoprotein receptor. Using a combination of close and distant species genomic sequence comparisons coupled with *in vivo *and *in vitro *studies, we found that a functional cholesterol-sensing sequence motif arose and was fixed within a pre-existing enhancer in the common ancestor of anthropoid primates.

**Conclusion:**

Our study demonstrates one molecular mechanism by which ancestral mammalian regulatory elements can evolve to perform new functions in the primate lineage leading to human.

## Abstract 2

Analysis of primate-specific evolution of the LDL receptor enhancer demonstrates a molecular mechanism by which ancestral mammalian regulatory elements can evolve to perform new functions.

## Background

Since King and Wilson's provocative paper was published in 1975 [1], differences in gene regulatory sequences have been predicted to be among the major sources of phenotypic evolution and divergence among animals. Consistent with this hypothesis, cis-regulatory changes have been found to play an important role in the evolution of morphologic features in model organisms [2]. In contrast, evolution of physiology has been linked to changes in protein coding sequences, when studied in animal vision, digestive metabolism, and host defense [3-7]. The contribution of regulatory sequence changes to the evolution of physiologic differences, however, is largely unexplored [8,9].

To examine the role of cis-regulatory changes in the emergence of novel physiologic traits in primates, we investigated the evolution of regulatory elements of the low-density lipoprotein (LDL) receptor gene (*LDLR*), which is a key player in maintaining lipid homeostasis. Cholesterol metabolism in humans has diverged in a variety of ways from that of many distant mammals such as rodents and dogs, with humans in general being more susceptible to diet-induced hypercholesterolemia [10]. The pivotal role of *LDLR *in cholesterol metabolism, coupled with its known expression differences among mammals [11], makes it a prime candidate for investigating primate-specific evolution of regulatory sequences. Here, we present molecular data supporting the gain of a cholesterol-sensing DNA motif in an ancestral mammalian *LDLR *regulatory element at a specific stage in primate evolution.

## Results and discussion

### Identification of primate-specific noncoding elements in the *LDLR *locus

**Figure 1:**
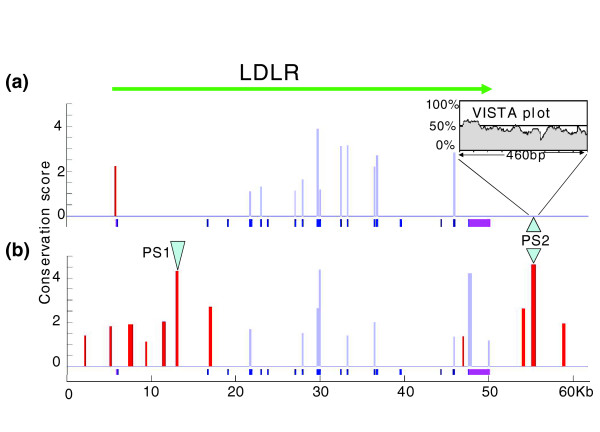
Conservation profiles of the *LDLR *locus using close (primate) and distant (human-mouse) species comparisons. **(a) **Human-mouse and **(b) **multiple primate (human, baboon, colobus, dusky titi, marmoset, and owl monkey) conservation profiles were calculated using Gumby and visualized using RankVISTA (see Materials and methods) and displayed with the human sequence as reference. Only about 6 kilobases (kb) of the 5' intergenic region is shown because of incomplete primate sequence availability. The entire 3' intergenic region was included in the analysis. Vertical bars depict conserved exonic (light blue) and nonexonic (red) sequences, with height indicating statistical significance of sequence conservation (see Materials and methods). *LDLR *coding exons (dark blue) and untranslated regions (UTRs; magenta) are marked below the conservation plots. Arrows denote the two highest-scoring primate-specific elements (PS1 and PS2). The inset shows the human-mouse VISTA plot for element PS2, with the vertical axis representing sequence identity calculated over a 100 base pair (bp) window.

To identify putative primate-specific *LDLR *regulatory sequences, we examined orthologous regions from a panel of mammals closely and distantly related to human for the presence of evolutionarily conserved noncoding sequences using Gumby, an algorithm that detects sequence blocks evolving significantly more slowly than the local neutral rate (see Materials and methods, below) [12-14]. Because humans and nonhuman primates share many features of cholesterol metabolism, we specifically scanned for elements that are preferentially conserved in primates under the hypothesis that primate-specific regulatory sequences contribute to the distinctive biology of those species. We conducted pair-wise sequence comparisons of the 83 kilobase (kb) genomic region containing *LDLR *and its entire 5' and 3' intergenic regions between human and each of a panel of distantly related species consisting of the prosimian lemur, mouse, and dog. In these comparisons we identified either the known promoter sequence alone (Figure 1a and data not shown) or a limited number of noncoding elements (Additional data file 1 and data not shown). The promoter region was the only noncoding region consistently identified as being conserved in the three pair-wise comparisons. In contrast, multiple sequence comparisons between human and a set of five anthropoid primate species, chosen on the basis of their evolutionary relationship using the 'phylogenetic shadowing' strategy [15], identified two human noncoding DNA elements, named PS (primate specific) 1 and 2, which were found to be highly significantly conserved (P approximately 10^-5^) in primates (Figure 1b). However, they were undetected in comparisons involving human and each of the distant species (Figure 1 and Additional data file 1).

To confirm independently the lack of significant conservation of the PS1 and PS2 elements between human and distant mammals, we also analyzed human-mouse alignment using a sliding-window percentage identity conservation criterion. We found that the human-mouse percentage identities across PS1 and PS2 were below 50% (Figure 1 and data not shown). This is close to the background percentage identity in aligned intergenic DNA and is well below the threshold of 70% identity that is normally applied to the detection of conserved functional sequences [16]. We further verified that the phastCons program [17] detects no conserved sequences overlapping PS1 and PS2 (data not shown). Although the phastCons predictions, obtained from the UCSC Genome Browser, are in general based on alignment of 17 mammalian and nonmammalian species, conservation scores in the *LDLR *locus reflect only mammalian conservation because more distant genomes exhibit very limited nonexonic alignment in this locus.

To assess quantitatively the conservation level of PS1 and PS2 between human and distant mammals, we identified the orthologous aligned counterparts of the human PS1 and PS2 elements in lemur, mouse, and dog. Gumby analysis of conservation scores indicated that each of these nonanthropoid primate sequences exhibited a level of similarity to the human sequence consistent with unconstrained evolution at the neutral rate (conservation *P *value; Table 1). Together, these analyses strongly suggest a lack of significant sequence constraint between the anthropoid primate and mammalian PS1 and PS2 sequences.

### The human *LDLR *PS2 element exhibits significantly greater enhancer activity than its mammalian orthologs

**Figure 2:**
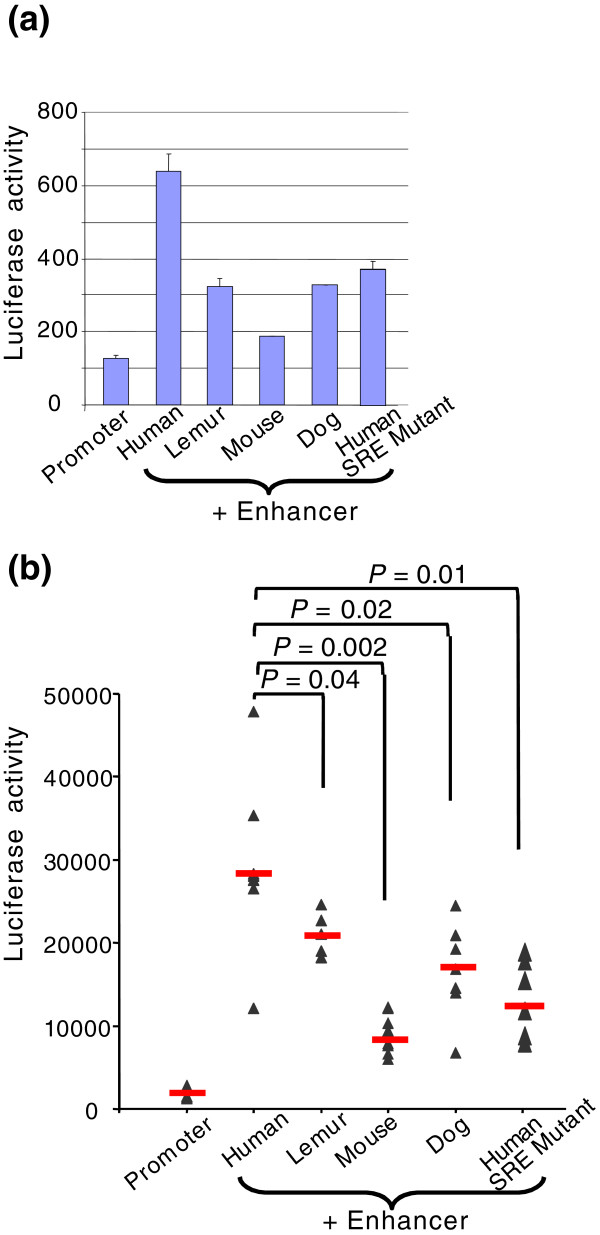
Human *LDLR *PS2 enhancer exhibits significantly higher activity than orthologous lemur, mouse, and dog enhancers. Luciferase assay analysis of **(a) **transient transfections into human 293T cells and **(b) **plasmid DNA transfer into mouse liver. The luciferase reporter constructs tested are either the *LDLR *promoter alone (promoter) or the promoter in combination with the *LDLR *PS2 enhancer from one of the indicated species. Error bars indicate standard deviation. 'SRE mutant' refers to the mutagenized human sterol regulatory element (SRE) with four point substitutions relative to the wild-type (WT) SRE (Figure 4a). Luciferase activity is reported in arbitrary units. Each triangle in panel b represents luciferase activity in an individual mouse. Red bars denote the median activity of each construct.

To explore the potential regulatory function of these two primate-specific conserved elements, we examined their ability to drive reporter gene expression in both a transient transfection assay in human 293T cells and in an *in vivo *mouse liver gene transfer assay [18]. Each human element plus approximately 200 base pairs (bp) of flanking sequence on either side was cloned upstream of the human *LDLR *promoter [19] fused to a luciferase reporter gene. Human element PS2, but not PS1, consistently increased luciferase expression approximately fivefold relative to the human promoter alone in both the *in vitro *and *in vivo *assays (Figure 2). The human element PS2 also increased luciferase expression when cloned upstream of the generic SV40 promoter, albeit to a lesser extent (twofold; Additional data file 3). Enhancer activity of this element was further confirmed by the finding that genomic region corresponding to PS2, but not PS1, is a DNaseI hypersensitive site in human liver cells (Additional data file 2 and data not shown).

To explore the regulatory function, if any, of mammalian sequences orthologous to human PS2, we cloned the PS2-aligned sequences from lemur, mouse, and dog into the luciferase reporter vector described above and compared their activities with that of the human sequence. Despite the lack of statistically significant sequence constraint between the human enhancer and its lemur, mouse, and dog orthologs, the latter three sequences exhibited enhancer activity both *in vitro *and *in vivo *(Figure 2). The human regulatory element, however, consistently exhibited stronger enhancer activity in both assays, driving twofold greater expression than lemur or dog PS2 and fourfold greater expression than mouse (Figure 2a). This observation, coupled with the evidence of negative selection acting on the primate enhancer and the lack of significant sequence constraint between the anthropoid primate and mammalian PS2 sequences (conservation *P *value; Table 1), suggests that the stronger enhancer activity in human is a gain of function in the anthropoid primate lineage with a potentially important adaptive role in these species.

### An anthropoid-primate specific sterol regulatory element contributes to distinct human PS2 enhancer activity

**Figure 3:**
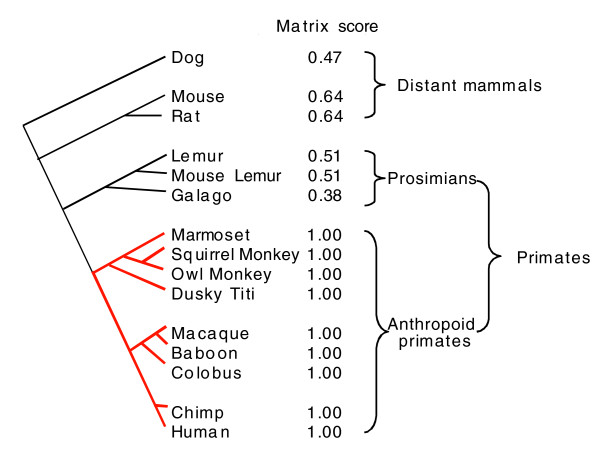
Phylogenetic analysis of the SRE. The human sterol regulatory element (SRE) motif and its orthologs were scored for transcription factor binding affinity, with low motif scores indicating low predicted affinity to SRE binding protein (SREBP; see Materials and methods). Because the SRE is present in all the analyzed anthropoid primates (indicated by the red branches in the tree) and absent from the prosimians, rodents, and dog, emergence in the lineage leading to anthropoid primates is the most parsimonious explanation.

To identify the molecular basis of the primate-specific activity of PS2, we computationally dissected the 860 bp human PS2 enhancer (see Materials and methods, below) and found a sterol regulatory element (SRE). This is a binding site specifically recognized by the cholesterol sensing proteins SREBPs (sterol regulatory element binding proteins), which are known to play a key role in the regulation of *LDLR *[20,21]. Phylogenetic analysis of the orthologous PS2 sequences from three distant mammals (mouse, rat, and dog), three prosimians (lemur, mouse lemur and galago), and nine anthropoid primates covering all major lineages including hominoids, and old-world and new-world monkeys revealed the presence of the SRE exclusively in anthropoid primates (Figure 3). This phylogenetic distribution of the SRE in mammals can most parsimoniously be explained by the appearance of the SRE in the ancestor of anthropoid primates after its divergence from prosimians (Figure 3).

The functional role of the binding motif identified by computational analysis was explored by site-specific mutagenesis. A 4 bp substitution was introduced into the SRE, which was expected to inactivate the site completely based on a previously reported mutagenesis study [22]. The 4 bp substitution in the SRE decreased human enhancer activity in the human cell culture assay and the *in vivo *mouse liver DNA transfer assay to a level comparable with that in lemur, mouse, and dog enhancers; these species lack a computationally predicted SRE (Figure 2). The functionality of the SRE, found exclusively in anthropoid primates, suggests that this element is likely to contribute to the stronger activity found in these species. We also identified within the 860 bp enhancer a 21 bp subregion that exhibits strong conservation across mammalian species including lemur, mouse lemur, galago, mouse and dog, and that contains predicted binding sites for transcription factors activating enhancer binding protein (AP)-4 and AP-1. Deletion of the conserved 21 bp sequence from either human or dog PS2 resulted in a significant reduction in enhancer activity (data not shown), suggesting that the evolutionarily conserved AP-4 and AP-1 sites are important for the core enhancer activity shared among mammals. It is worth noting that such short blocks of genuinely constrained sequence are not easily distinguishable from the numerous 'coincidentally conserved' sequence fragments that are likely to occur in large genomic regions as a consequence of stochastic variation in the incidence of neutral mutations. Incorporation of additional information, namely the binding specificities of transcription factors, was required to classify this 21 bp fragment as a functional candidate. Thus, conservation of this short subsequence in multiple mammals does not detract from the fact that the enhancer sequence is significantly conserved only in anthropoid primates, as described above.

**Figure 4:**
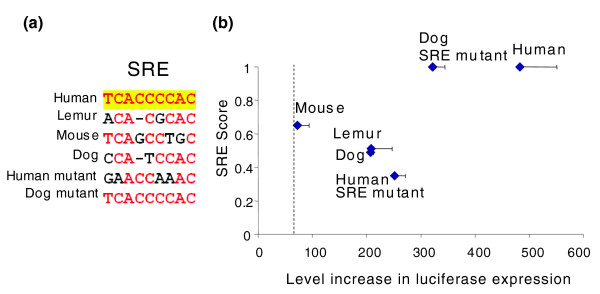
Relation between SRE motif score and response of PS2 enhancer to SREBP-2. **(a) **Alignment of sequences from indicated species at positions orthologous to human PS2 sterol regulatory element (SRE). 'Human mutant' refers to the mutated human SRE with four point substitutions relative to the human wild-type. 'Dog mutant' refers to the consensus SRE introduced into dog PS2 by means of three point substitutions. **(b) **Luciferase assay analysis of reporter construct and the SRE binding protein (SREBP)-2 expression vector cotransfection into 293T cells. The *LDLR *PS2 element from each of the indicated species was tested in combination with the *LDLR *promoter. The Y-axis denotes SRE motif score (likeness to known SRE motifs; see Materials and methods). Response of the PS2 element to SREBP-2 is shown as the increase in luciferase expression level (arbitrary units) on activation by 3 ng SREBP-2 expression vector. Expression level increase for *LDLR *promoter alone is indicated by the dotted line. Error bars indicate standard deviation.

Because SREBP-2, the major regulator of LDLR [20,21], specifically binds to the SRE [11], we examined the responsiveness of the human, lemur, mouse, and dog orthologous PS2 enhancers to this transcription factor. Co-transfection of the reporter gene driven by PS2 and the human *LDLR *promoter with a construct expressing the mature form of SREBP-2 indicated that the human enhancer was strongly activated by the exogenous SREBP-2, to a level fivefold higher than that of the human *LDLR *promoter alone, which is known to be SREBP responsive as well [23]. The lemur, mouse, and dog enhancers were activated to a significantly lesser extent, which is consistent with their much lower SRE prediction score and with their lack of additional consensus SRE motifs within the PS2 element (Figure 3, Figure 4a, and data not shown). To determine whether the observed differential SREBP-2 response among tested mammalian PS2 enhancers was directly mediated by the predicted SRE, we inactivated or restored the consensus SRE by site-specific mutagenesis at the orthologous positions of the human and dog PS2 element, respectively. Substituting four bases in the human SRE motif, which reduced the motif matrix score from 1 to 0.35 (see Materials and methods, below), resulted in a reduction in SREBP-2 enhancer response to a level comparable to that of the lemur, mouse, and dog enhancers. These results indicate that the anthropoid-specific SRE mediates the activation of the PS2 enhancer by SREBP-2 and contributes to the strong enhancer activity characterizing human and other anthropoid primates. Furthermore, substituting three bases in the dog SRE, so as to increase the SRE motif score from 0.47 to 1 (representing a perfect SRE), led to a significant increase in the dog enhancer response to SREBP-2, although only to half the level of the human PS2 enhancer (Figure 4b). This suggests that the anthropoid primate-specific SRE is part of a combinatorial mechanism [24], including possible additional substitutions in the core enhancer element that contribute to the stronger human PS2 enhancer activity.

**Figure 5:**
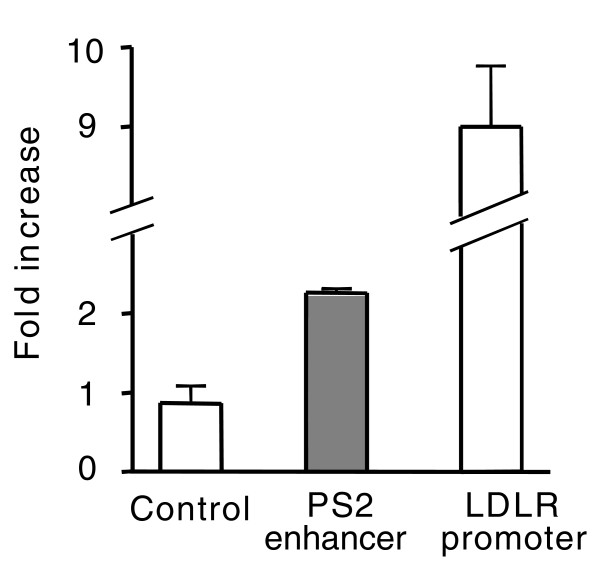
Anti-SREBP-2 antibody specifically enriches human PS2 DNA sequences in HepG2 cells in a ChIP assay. DNA precipitates were measured by real-time polymerase chain reaction using primers spanning the indicated regions. Control region (control) corresponds to the first coding exon of the neighboring gene *SPBC24*, which is approximately 7.5 kilobases away from the PS2 sequence. The results are presented as fold increase in the enrichment of precipitated DNA by anti-SREBP-2 (anti-sterol regulatory element binding protein-2) antibody over nonspecific IgG. Graphical representations of the mean ± standard error from three independent experiments are shown.

The role of SREBP-2 in regulating the human PS2 enhancer was further explored in its native chromosomal context in HepG2 cells, which actively express SREBP-2 and are a well defined system for studying *LDLR *regulation [25-27]. Our analysis showed that the PS2 sequence is a DNaseI hypersensitive site in HepG2 cells (Additional data file 2), suggesting that the corresponding DNA element is involved in transcriptional regulation of the endogenous gene. Using the ChIP (chromatin immunoprecipitation) assay, we were able to show that fractionation of chromatin with an anti-SREBP-2 antibody specifically enriched for endogenous PS2 and *LDLR *promoter DNA relative to control region (Figure 5); the latter has previously been shown to be bound by SREBP-2 [28]. Together, the DNAseI hypersensitivity and ChIP assays provide strong evidence that SREBP-2 binds in the vicinity of the human PS2 enhancer in its native genomic locus. Regulation of the enhancer by SREBP-2 also suggests that the PS2 element plays a role in the activation of its upstream gene *LDLR *rather than the downstream gene Spbc24, which encodes a component of the kinetochore Ndc80 protein complex [29]. It was recently noted, based on genome-wide analysis of gene expression, that SREBP targets are largely restricted to lipid metabolism genes, including *LDLR *[20]. No connection was found between SREBP and kinetochore structural genes such as *Spbc24*.

## Conclusion

We have shown phylogenetic and molecular data supporting the evolution of differential gene expression of *LDLR *in mammals. Transcriptional control of *LDLR *is mainly effected through the intracellular cholesterol sensor SREBP-2. The latter was previously shown to mediate the increased transcription of *LDLR *in response to low cholesterol levels through an SRE in the *LDLR *promoter [23,30], which is conserved in all mammals examined. The additional SRE found in the PS2 enhancer in primates may lead to differential response to SREBP-2 among mammals. Although the contribution of the PS2 enhancer to the *in vivo *regulation of LDLR remains to be elucidated, these results suggest that species-specific regulation of *LDLR *is expected in conditions that result in decreased intracellular cholesterol levels, such as reduced availability of dietary cholesterol, and has implications for the study of *LDLR *response to cholesterol-lowering drugs in animal models.

Although the human *LDLR *coding sequence and promoter are well conserved in all sequenced mammals (Figure 1 and Additional data file 1), our data support the modification of the expression characteristics of this gene through the primate-specific evolution of a distal regulatory element. We have shown the emergence and fixation of a SRE in the common ancestor of anthropoid primates, which modifies the expression driven by a pre-existing mammalian enhancer shared by all tested mammals. This demonstrates one mechanism by which mammalian regulatory elements can evolve to perform new functions. Given the vital importance of LDLR in energy storage, the appearance of a new cholesterol sensing element in the LDLR enhancer might have played a role in the evolution of new physiologic features, because the ancestor of anthropoid primates adapted to different metabolic requirements and diets.

## Materials and methods

### Plasmid constructs

The human LDLR promoter was cloned in the proper orientation upstream of the luciferase cDNA in the pGL3Basic construct (Promega, Madison, WI, USA). The human PS1 element and the PS2 elements from human, lemur, mouse, and dog LDLR loci were polymerase chain reaction (PCR) cloned into poly-linker sites in the (-) orientation upstream of the promoter. The cloned human PS1 element corresponds to the Gumby predicted conserved sequence and approximately 200 bp of flanking sequence on either side (hg18, chr19:11067913-11068639). To clone the human PS2 element, the region containing human PS2 was PCR cloned into pGL3Basic (see Additional data file 4 for primer sequences), and digested with *Spe*I and *Nhe*I to only include the Gumby predicted conserved sequence and approximately 200 bp of flanking sequence on either side (hg18, chr19:11110333-11111194). Site-specific point mutations and deletions were introduced into human and dog PS2 elements using QuikChangeII site-directed mutagenesis kit (Stratagene, La Jolla, CA, USA), in accordance with the manufacturer's protocol and were confirmed by sequencing. The expression construct for human mature form of SREBP-2 (pcDNA.2FLAG SREBP-2) was kindly provided by Dr Timothy F Osborne (UC Irvine). The human LDLR promoter (hg18, chr19:11060880-11061099) was PCR amplified (see Additional data file 4 for primers used).

### Transient transfection reporter assay

Cells of human embryonic kidney cell line 293T (ATCC CRL-11268) were grown at 37°C and 5% carbon dioxide CO2 in Dulbecco's modified Eagle's medium (ATCC), supplemented with 10% fetal bovine serum (Hyclone, Logan, UT, USA), l-glutamine, and penicillin-streptomycin. Cells with a passage number below 15 were used. The cells were grown in 12-well plates (4 × 10^4 ^cells/well) and transfected using Fugene (Roche Molecular Biochemicals, Indianapolis, IN, USA), in accordance with the manufacturer's protocol. Briefly, 100 ng of each assayed plasmid and 10 ng pCMVβ (BD Biosciences, Franklin Lakes, NJ, USA) were mixed with 1.5 μl Fugene and added to each well. Following 42-48 hours of incubation, cells were harvested and lysed. Activity of luciferase and β-galactosidase was measured using the Luciferase Assay System (Promega) and the Galacto-Light Plus (Applied Biosystems, Foster City, CA, USA), respectively. Luciferase activity for each sample was normalized to the β-galactosidase assay control. For co-transfection experiments, 100 ng of the lulciferase reporter gene construct, 3 ng of SREBP-2 expression vector, and 10 ng of pCMVβ were used. Transfections were carried out in duplicate. All experiments are representative of at least three independent transfections.

### Tail vein plasmid DNA transfer assays

Tail vein injection was performed as described by Herweijer and Wolff [18], following the TransIT^® ^*In Vivo *Gene Delivery System (Mirus Corporation, Madison, WI, USA) protocol. Six to nine FVB male mice (Charles River Laboratory, Wilmington, MA, USA) at age 7-8 weeks were used for each reporter gene construct. Ten micrograms of each reporter construct, along with 2 μg of pCMVβ (BD Biosciences) to correct for delivery efficiency, were injected into each mouse. The entire content of the syringe was delivered in 3-5 s. Animals were killed 24 hours later, livers extracted, measured to correct for size, homogenized, and centrifuged for 15 min at 4°C, 14,000 rpm. Activities of luciferase and β-galactosidase were measured as described above. All *P *values are from the two-sample Wilcoxon rank-sum (Mann-Whitney) test using STATA (STATA Corporation, College Station, TX, USA). All experimental results are representative of two independent plasmid DNA transfer assays.

### Chromatin immunoprecipitation

HepG2 cells (ATCC HB-8065) were cultured in DMSF (defined medium serum free) medium for 24 hours for induction of endogenous SREBPs [31]. Chromatin immunoprecipitaion assays were performed as described previously [32]. Cross-linked chromatin was immunoprecipitated with a specific SREBP-2 antibody (Santa Cruz sc-8151) [8] or IgG control.

### Sources of sequence data

Draft sequences of baboon, colobus, dusky titi, marmoset, owl monkey, lemur, and galago bacterial artificial chromosomes (BACs) were determined by sequencing ends of 3 kb subclones to 8- to 10-fold coverage using BigDye terminators (Applied Biosystems) and assembling reads into contigs with the Phred-Phrap-Consed suite, as described previously [33]. All BAC sequences were submitted to GenBank with the following species and accession numbers: baboon (*Papio hamadryas*), AC140974; colobus (*Colobus guereza*), AC150433; marmoset (*Callithrix jacchus*), AC145530; dusky titi (*Callicebus moloch*), AC144655; owl monkey (*Aotus hybrid*), AC171393; squirrel monkey (*Saimiri boliviensis boliviensis*), AC146467; lemur (*lemur catta*), AC118569; mouse lemur (*microcebus murinus*), AC175656; and galago (*otolemur garnetti*), AC175655). Human, chimpanzee, rhesus, mouse, rat, and dog sequences were downloaded from the UCSC Genome Bioinformatics website [34].

### Analysis of sequence conservation

We aligned the human *LDLR *locus (chr19:11,055,219-11,117,169; NCBI Build 35) to its orthologs in baboon, colobus, dusky titi, marmoset, owl monkey, lemur, mouse, and dog using MLAGAN [35]. Because incomplete primate sequence availability, we included only about 6 kb of the 5' intergenic region in the analysis. Aligned sequences were scanned for statistically significant evolutionarily conserved regions using Gumby [12-14]. Gumby goes through the following three-step process to identify statistically significant conservation in the global alignment input. First, noncoding regions in the alignment are used to estimate the local neutral mismatch rates among all pairs of aligned sequences. The rates are used to derive a log-likelihood scoring scheme for slow versus neutral evolution, in which the slow rate is set to some fraction (in this case, half) of the neutral rate. Second, each alignment position is then assigned a conservation score using a phylogenetically weighted sum-of-pairs scheme. Third, conserved regions of any length are identified as alignment blocks with a high cumulative conservation score and assigned *P *values using Karlin-Altschul statistics [36]. We set a threshold *P *value of 0.1 in a baseline human sequence length of 10 kb. Conserved regions identified by Gumby were visualized using RankVISTA. In addition, human-mouse sequence conservation was analyzed using the VISTA web server [37,38], with the standard criterion of 70% sequence identity in window of size 100 bp.

### Binding site prediction

We scanned the aligned enhancer sequences for predicted transcription factor binding sites using DiAlign TF [39]. Anthropoid primate (human, baboon, colobus, dusky titi, marmoset, and owl monkey) sequences were assessed for the presence of sites conserved across all six species that were predicted to bind one of the following liver-expressed transcription factors: C/EBP (CCAAT/enhancer-binding protein), LXR (liver X receptor), FXR (farnesoid X receptor), COUP-TF (Chicken Ovalbumin Upstream Promoter Transcription Factor), peroxisome proliferator-activated receptor (PPAR), HNF1 (hepatocyte nuclear factor 1), HNF3, HNF4, and SREBP. Binding sites common to primates and mammals were predicted on the basis of conservation of any vertebrate transcription factor motif in at least eight of the 10 analyzed species (six anthropoid primates, lemur, mouse, rat, and dog). Motif scores of the SREs or SRE orthologs of individual species were calculated using rVISTA [40] and normalized so that the maximum achievable score is 1.0, and the expected score of a random nucleotide sequence with the local GC content is zero. The score distribution of functional SREs was calculated from the binding profile of SREBP [41], assuming that nucleotide frequencies at each position in the motif are independent. We retrospectively augmented the species set with SRE orthologs from chimpanzee, rhesus, squirrel monkey, mouse lemur and galago, based on pair-wise alignments of those species to human.

## Additional data files

The following additional data are available with the online version of this paper: A figure showing the conservation profiles of the *LDLR *locus using (A) human-dog and (B) human-lemur sequence comparisons (Additional data file 1); a figure showing the DNaseI hypersensitive site mapping around the *LDLR *PS2 region in human liver cell line HepG2 (Additional data file 2); a figure showing that human *LDLR *PS2 enhancer activity is independent of the *LDLR *promoter (Additional data file 3); a table listing the primers used in the cloning of human *LDLR *promoter and PS2 elements from indicated species (Additional data file 4); and a table listing the primers used in ChIP assay (Additional data file 5).

## Supplementary Material

Additional data file 1 A figure showing the conservation profiles of the *LDLR *locus using (A) human-dog and (B) human-lemur sequence comparisons. Click here for file

Additional data file 2 A figure showing the DNaseI hypersensitive site mapping around the *LDLR *PS2 region in human liver cell line HepG2. Click here for file

Additional data file 3 A figure showing that human *LDLR *PS2 enhancer activity is independent of the *LDLR *promoter. Click here for file

Additional data file 4 A table listing the primers used in the cloning of human *LDLR *promoter and PS2 elements from indicated species. Click here for file

Additional data file 5 A table listing the primers used in ChIP assay. Click here for file

## Figures and Tables

**Table 1:** PS2 enhancer functional divergence correlates with sequence constraint

Sequence analysis			Functional test		
Species compared	Conservation *P *value		Species assayed	Relative enhancer strength	
			
	PS1	PS2		PS1	PS2

Human/5 primates	4.8 × 10^-5^	10^-5^	Human	0.9	5.1
Human/lemur	~1	0.76	Lemur	ND	2.6
Human/mouse	~1	~0.99	Mouse	ND	1.5
Human/dog	0.28	0.45	Dog	ND	2.6

Conservation *P *values are calculated using Gumby [12] under the null hypothesis of evolution at the neutral (background) rate. Low *P *values indicate that the null model of neutrality should be rejected, with the lowest *P *values identify the most significantly conserved sequences. The sequences analyzed for human-mammal conservation or enhancer activity correspond to the Gumby predicted conserved sequence and approximately 200 base pairs of flanking sequence on either side (see Materials and methods). Enhancer strength is shown as fold increase over promoter alone in luciferase assays in 293T cells. ND, not done.
