# Supplementary material for: Primate-specific evolution of an LDLR enhancer
Source: Genome Biol. 2006 Aug 2;7(8):R68. doi: 10.1186/gb-2006-7-8-r68 (PMC1779597; doi:10.1186/gb-2006-7-8-r68)
Supplement: Additional data file 4 — A table listing the primers used in the cloning of human LDLR promoter and PS2 elements from indicated species. [file gb-2006-7-8-r68-S4.pdf]

Supplemental Table 1: Primers used in the cloning of human *LDLR* promoter and PS2 elements from indicated species.

Element ID	Primers
Human LDLR promoter	F: ATGCGTTTCCAATTTTGAGG<br>R: TCTAGCAGGGGGAGGAGTTT
Human PS1	F: AGCCTCAGTCATGCCACTG<br>R: GGCCTAGGCAACATACCAAG
Human PS2	F: GGAGGCCACTGTGTCAGTTT<br>R: ACTCCAGCCTGGGAAACTCT
Lemur PS2	F: ATCCCCAGCTGGTATCCTCT<br>R: ACAGCTGGTTTCACAGCATT
Mouse PS2	F: GCAGCAGCTGATTTCTGACA<br>R: AGGCATGCTTGTGAGAGGTA
Dog PS2	F: TTGGTACCCCCAACTCTGTC<br>R: CAGAGCCAGGATTTGACCTG
HumanPS2 21-bp deletion:	CATCCCAGACCACCTGGAGCCTCTG
DogPS2 21-bp deletion:	GGCGGCCCAAACGCTGGAGCCTGC
HumanPS2 SRE mutant:	GTCCCTGGTCCCCAGAACCAAACTGAGCATGGCCGC
DogPS2 SRE mutant:	GCCCTGGGCACATCACCCCACCCCGAGCGGGGC
