# Supplementary material for: Primate-specific evolution of an LDLR enhancer
Source: Genome Biol. 2006 Aug 2;7(8):R68. doi: 10.1186/gb-2006-7-8-r68 (PMC1779597; doi:10.1186/gb-2006-7-8-r68)
Supplement: Additional data file 5 — A table listing the primers used in ChIP assay. [file gb-2006-7-8-r68-S5.pdf]

Supplemental Table 2: primers used in ChIP assay

Region	Primers
Human PS2	F: TCTGAGTGGGAGTCCCTGGT<br>R: GAAAGTTGCCAGGAAACCCC
Human LDLR promoter	F: AATGACGTGGGCCCCG<br>R: ACCTGCTGTGTCCTAGCTGGA
Control	F: TGATGAATTTCCTGGAGAGCTG<br>R: TCCATCATGGCCCCAGTG
